# The Homœopathic Medical Doctrine; or, "Organon of the Healing Art:" A New System of Practice

**Published:** 1833-10

**Authors:** 


*The Homœopathic Medical Doctrine; or, "Organon of the Healing Art:" a new System of Physic.* Translated from the German of S. HAHNEMANN, by CHARLES H. DEVRIENT, Esq.; with Notes, by SAMUEL STRATTEN, M.D. Dublin, 1833. 8vo pp. 332.

THE homœopathic system is founded upon two axioms. The first is, that all diseases are to be cured by the remedies which would produce those diseases in healthy persons; the second is, that these medicines are to be given in infinitesimal doses. Dr. Hahnemann, the inventor of this theory, or, as he would say, the discoverer of these facts, has propagated his system in Germany with unwearied zeal for nearly forty years; but we do not recollect that it was mentioned in England before the appearance of an able article on the subject, in the Edinburgh Review, in 1827. Since that time occasional remarks on Homœopathy have appeared in the medical journals; and now, at last, we have a translation of the "Novum Organon," from the pen of Mr. Devrient. The German copy of this book, which is now lying before us, is dated 1824, and, by comparing it with Mr. Devrient's translation, we find that Dr. Hahnemann has made considerable additions to his work since that period; yet, whenever we found anything incomprehensible in Mr. D.'s book, we referred to our Hahnemann, and we found that either Hahnemann must have wilfully changed sense into nonsense since 1824, or that Mr. Devrient must have committed a blunder: we incline to the latter theory. Thus the translator, at p. 92, makes his author say, "If we except all these cases, we shall find that those which they have cured promptly and permanently, by the bounty of Providence alone, are, to the mass of their other irrational cures, in the proportion of one to a thousand." But, in our German copy, (p. 45,) Hahnemann talks of "mehren hunderten solcher elenden Curen;" *i. e.* of hundreds of such wretched methods of treatment, not cures. We shall have occasion to mention some other mistranslations in the course of our analysis.

Dr. Hahnemann commences his introduction with what he calls "an examination into the allopathic doctrines that have existed till the present time." These doctrines do not meet with any mercy at his hands; they are called "vague dreams, gratuitous suppositions, hypotheses destitute of foundation, skilfully invented for the convenience of therapeutic medicine," &c.; and, after a thousand censures of the practice of the old school, he proceeds to say,

"The old school practise yet another method of cure, which they call 'exciting and strengthening' (by *excitantia*, *nervina*, *tonica*, *confortantia*, *roborantia*.) It is surprising that they should boast of this mode of treatment.

"Has it ever succeeded in removing the weakness which a chronic disease so often engenders, augments, and keeps up, by prescribing (as it has so frequently done) light Rhine wine, or Tokay? As this treatment was not able to cure the chronic disease, (the source of the debility,) the strength of the patient decreased in proportion as they made him take more wine, because the vital powers, in their re-action, oppose relaxation to artificial excitements.

"Did cinchona, or any of the substances which collectively bear the name of *Amara*, ever restore strength in these cases which are of such frequent occurrence? These vegetable products, which they pretended were tonic and strengthening in all circumstances, together with the preparations of iron, did they not add fresh sufferings to the old ones, by reason of their peculiar morbific action, without being able to remove the debility which depended on an unknown malady of long standing?" (P. 40.)

We rather think that the allopathists will venture to answer "yes" to both these sturdy questions. He also ventures to assert that "the causes of disease cannot possibly be material, since the least foreign substance introduced into the blood-vessels, however mild it may appear to us, is suddenly repulsed by the vital power, as a poison; or, where this does not take place, death itself ensues;" and adds, that a little pure water injected into the veins destroys life. But it is needless to contradict these phantasies; and we therefore pass on with pleasure to the next section, in which Dr. Hahnemann is much stronger: it is entitled "Examples of Homœopathic Cures, performed unintentionally by physicians of the old school of medicine." The old school made use of "counteroperating remedies and palliatives, according to the method *contraria contrariis*." But this is just the reverse of what they ought to have done.

"Observation, reflection, and experience, have unfolded to me that the best and true method of cure is founded on the principle *similia similibus curentur.* To cure in a mild, safe, and durable manner, it is necessary to choose in each case a medicine that will excite an affection similar (ὁμοιον πάθος) to that against which it is employed.

"Until the present time no person has ever inculcated this homœopathic mode of treatment, and, yet more, no one has ever put it into practice. But, if this is the only true method, (of which every one may be convinced with myself,) we ought to discover sensible traces of it in every epoch of the art, although its true character may have been unknown during thousands of years; and such has in reality been the case." (P. 48.)

The following are among the instances of homœopathic cures given by our author; and it must be confessed that they form a body of evidence which is rather staggering. We regret that we cannot extract them all.

"The author of the treatise on epidemic diseases (attributed to Hippocrates,) mentions a case of cholera morbus that resisted every remedy, and which he cured by means of white hellebore alone, which, however, excites cholera of itself, as witnessed by Forestus, Ledelius, Reimann, and many others.

"The English sweating sickness which first exhibited itself in the year 1485, and which, more murderous than the plague itself, carried off in the commencement, (as testified by Willis,) ninety-nine patients out of a hundred, could not be subdued until such time as they had learned to administer sudorifics to patients. Since that time, as Sennertus observes, few persons died of it.

"A case of dysentery which lasted several years, threatening the patient with inevitable death, and against which every other medicine had been tried without success, was, to the great surprise of Fischer, (but not to mine,) cured in a speedy and permanent manner by a purgative administered by an empiric.

"Murray, (whom I selected from numerous other authorities,) together with daily experience, inform us, that among the symptoms produced by the use of tobacco, those of vertigo, nausea, and anxiety are the principal. Whereas Diemerbroeck, when attacked with those very symptoms of vertigo, nausea, and anxiety, in the course of his close attendance on the victims of epidemic diseases in Holland, removed them by the use of the pipe.

"The hurtful effects which some writers (among others, Georgi) ascribe to the use of the *agaricus muscarius*, by the inhabitants of Kamtschatka, and which consist of tremors, convulsions, and epilepsy, became a salutary remedy in the hands of C. G. Whistling, who used this mushroom with success in cases of convulsions accompanied with tremor; likewise in those of J. C. Bernhardt, who used it with success in a species of epilepsy.

"The remark made by Murray, that oil of aniseed allays pains of the stomach and windy colic caused by purgatives, ought not to surprise us, knowing that J. P. Albrecht has observed pains in the stomach produced by this liquid, and P. Forestus violent colic likewise caused by its administration.

"If F. Hoffman praises the efficacy of Millefoil in various cases of hemorrhage; if G. E. Stahl, Buchwald and Löseke, have found this plant useful in excessive hemorrhoïdal flux; if Quarin and the editors of the *Breslauer Sammlungen* speak of the cure it has effected of hemoptysis; and finally, if Thomasius (according to Haller) has used it successfully in uterine hemorrhage; these cures are evidently owing to the power possessed by the plant, of exciting of itself intestinal hemorrhage and hematuria, as observed by G. Hoffman, and more especially of producing epistaxis, as confirmed by Boecler." (P. 51.)

"Stoerck, who had so intimate a knowledge of medicines, was on the point of discovering that the bad effects of the dictamnus, which sometimes provokes a mucous discharge from the vagina, arose from the very same properties in this root by virtue of which he cured a leucorrhea of long standing.

"Stoerk might in like manner have been struck with the idea of curing a general chronic eruption, humid, phagedenic, and psoric, with the clematitis, having himself ascertained that this plant has the power of producing a psoric eruption over the whole body.

"If, according to Murray, the euphrasia cures a certain form of ophthalmy, how could it otherwise have produced this effect, but by the faculty it possesses of exciting a kind of inflammation in the eyes, as has been remarked by Lobel?

"According to J. H. Lange, the nutmeg has been found efficacious in hysterical fainting fits. The sole natural cause of this phenomenon is homœopathic, and can be attributed to no other circumstance but that the nutmeg, when given in strong doses to a man in health, produces, according to J. Schmid and Cullen, mental excitement and general insensibility.

"The old practice of applying rose-water externally in ophthalmic diseases, looks like a tacit avowal that there exists in the leaves of the rose some curative power for diseases of the eye.

This is founded upon the homœopathic virtue which the rose possesses, of exciting by itself a species of ophthalmia in persons who are in health, an effect which Echtius Ledel and Rau actually saw it produce.

"If, according to De Rossi, Van Mons, J. Monti, Sybel, and others, the poison sumac has the faculty of producing pimples which gradually cover the entire body, it may be easily imagined that this plant is capable of effecting an homœopathic cure of various kinds of eruptive diseases, which it really has done, according to information furnished by Dufresnoy and Van Mons. What could have bestowed upon the poison sumac (as in a case cited by Alderson,) the power of curing a paralysis of the lower extremities, attended with weakness of the intellectual organs, if it did not, of itself, evidently possess the faculty of depressing the muscular powers by acting on the imagination of the patient to such a degree, as to make him believe that he is at the point of death, as in a case witnessed by Zadig." (P. 56.)

Our German copy has it: "Eben so wenig durfte es Stoerck auffallen, wenn er mit Brenn-Waldrebe eine Art langwierigen, feuchten, fressendeu, allgemeinen, krätzartigen Ausschlags heilte," &c. *i. e.* "As little ought Stoerck to have been surprised at curing a kind of chronic, moist, phagedenic, general, itch-like eruption with the clematis, &c.; and not "Stoerck might in like manner have been struck," &c.

The following are the natural and homœopathic effects of belladonna and hyoscyamus:

"Among the effects which belladonna excites when administered to a person in sound health, are symptoms, which, taken collectively, present an image greatly resembling that species of hydrophobia caused by the bite of a mad dog, a disease which Mayerne, Münch, Buchholz, and Neimike, cured in a perfect manner with this plant. The patient in vain endeavours to sleep, the respiration is embarrassed, he is consumed by a burning thirst attended with anxiety; the moment any liquids are presented to him, he rejects them with violence; his countenance becomes red, his eyes fixed and sparkling, (as observed by F. C. Grimm;) he experiences a feeling of suffocation while drinking (according to E. Camerarius and Sauter); for the most part he is incapable of swallowing any thing (as affirmed by May, Lottinger, Sicelius, Buchave, D'Hermont, Manetti, Vicat, and Cullen;) he is alternately actuated by terror and a desire to bite the persons who are near him (as seen by Sauter, Dumoulin, Buchave and Mardorf;) he spits everywhere around him (according to Sauter;) he endeavours to make his escape (as we are informed by Dumoulin, E. Gmelin, and Buch'oz;) finally, convulsion of the entire frame ensues (as witnessed by Boucher, E. Gmelin, and Sauter.) Belladonna has also effected the cure of different kinds of madness and melancholy, as in the cases reported by Evers, Schmucker, Schmalz, the two Münch's, and many others, because it possesses the faculty of producing different kinds of insanity, like those noted by Rau, Grimm, Hasenest, Mardorf, Hoyer, Dillenius, and others. Henning, after vainly endeavouring, during three months, to cure a case of amaurosis with coloured spots before the eyes, by a variety of medicines, was at length struck with the idea that this malady might perhaps be occasioned by gout, although the patient had never experienced the slightest attack; and upon this supposition he was by chance induced to prescribe belladonna, which effected a speedy cure free from any inconvenience. He would undoubtedly have made choice of this remedy at the commencement, had he known that it was not possible to perform a cure but by the aid of a remedy which produces symptoms similar to those of the disease itself; and that, according to the infallible law of nature, belladonna could not fail to cure this case homœopathically, since, by the testimony of Sauter and Buchholz, it excites, of itself, a species of amaurosis with coloured spots before the eyes.

"The hyosciamus has cured spasms which strongly resembled epilepsy, as witnessed by Mayerne, Stoerck, Collin, and others. It produces this effect by the very same power that it excites convulsions similar to those of epilepsy, as observed in the writings of E. Camerarius, C. Seliger, Hünerwolf, A. Hamilton, Planchon, Acosta, and others.

"Fothergill, Stoerck, Hellwick, and Ofterdinger, have used hyosciamus with success in certain kinds of mental derangement. But the use of it would have been attended with equal success in the hands of many other physicians, had they confined it to the cure of that species of mental alienation which resembles a kind of stupefaction, that Van Helmont, Wedel, T. G. Gmelin, Laserre, Hünerwolf, A. Hamilton, Kiernander, J. Stedmann, Torzetti, J. Faber, and Wendt, saw produced by the action of this plant.

"By taking the effects of hyosciamus collectively, which the latter observers have seen it produce, they present a picture of hysteria arrived at a tolerable height. We also find in J. A. P. Gessner, Stoerck, and in the *Act. Nat. Cur.*, that a case of hysteria, which bore great resemblance to the above mentioned, was cured by the use of this plant.

"Schenkbecher would never have succeeded in curing a vertigo of twenty years' standing, if this plant did not possess in a very high degree the power of creating generally an analogous state, as attested by Hünerwolf, Blom, Navier, Planchon, Sloane, Stedmann, Greding, Wepfer, Vicat, and Bernigau.

"A man, who had become deranged through jealousy, was for a long time tormented by Mayer Abramson with remedies that produced no effect on him, when, under the name of a soporific, he one day administered hyosciamus, which cured him speedily. Had he known that this plant excites jealousy and madness in persons who are in health, and had he been acquainted with the homœopathic law, (the sole natural basis of therapeutics,) he would have been able to administer hyosciamus from the very commencement with perfect confidence, and thus have avoided fatiguing the patient with remedies which, not being homœopathic, could be of no manner of service to him.

"The mixed prescriptions which were employed for a long time with the greatest success by Hecker, in a case of spasmodic constriction of the eyelids, would have proved ineffectual, if some happy chance had not included hyosciamus, which, according to Wepfer, excites a similar affection in persons who are in sound health.

"Neither did Withering succeed in curing a spasmodic constriction of the pharynx, with inability to swallow, until he administered hyosciamus, whose special action consists in causing a spasmodic constriction of the throat, with the impossibility of swallowing; an effect which Torzetti, Hamilton, Bernigau, Sauvages, and Hünerwolf, have seen it produce in a very high degree." (P. 65.)

In like manner Hufeland cured a case of lethargy, and Tralles an obstinate constipation, with opium; and Rave and Wedekind suppressed uterine hemorrhage with savine; and the reason that the cow-pock prevents variola, is the similarity of the two diseases. So again, the imbecility and mental alienation which mercury is capable of producing, together with its salivating power, explain how G. Perfect was enabled by mercury to cure a case of melancholy, alternating with increased secretion of saliva. After a hundred similar instances, our author observes, that even in the practice of domestic medicine, the same great truth has sometimes been discovered by persons of sound judgment; as, for instance,

"Frozen sourcrout is frequently applied to a limb that is recently frozen, or sometimes it is rubbed with snow.

"A cook who has scalded his hand exposes it to the fire at a certain distance, without heeding the increase of pain which it at first occasions, because experience has taught him that, by acting thus, he can in a very short time perfectly cure the burn, and remove every feeling of pain.

"Other intelligent individuals, equally strangers to medical science, (such, for example, as the lacker-workers,) apply a substance to burns which excites of itself a similar feeling of heat,—that is to say, hot alcohol, or the oil of turpentine; and by these means cure themselves in a few hours, well knowing that the so-called cooling ointments would not produce the same result in an equal number of months, and that cold water would only make the evil worse." (P. 95.)

The physicians who have been quoted in the above extracts cured "unintentionally," to use Hahnemann's word; but occasionally some one arose who had a glimpse of the true theory of healing.

"Thus the author of the book περὶ τόπων τῶν κὰτ' ἄνθρωπον, which forms a part of the books attributed to Hippocrates, expresses himself in the following remarkable words: διὰ τὰ ὅμοια νοῦσος γίνεται, καὶ διὰ τὰ ὅμοια προσφερόμενα ἐκ νοσεύντων ὑγιαίνονται,—διὰ τὸ ἐμέειν ἔπετος παύεται.

"Physicians of a later period have likewise known and proclaimed the truths of homœopathy. Thus, Boulduc, for example, discovered that the purgative properties of rhubarb were the faculty by which this plant cured diarrhœa.

"Detharding guessed that the infusion of senna would cure the colic in adults, by virtue of the faculty which it possesses of exciting that malady in healthy persons.

"Bertholon informs us that, in diseases, electricity diminishes, and finally removes, a pain which is very similar to one which it also produces.

"Thoury affirms that positive electricity accelerates arterial pulsation; also that it renders the same slower, where it is already quickened by disease.

"Stoerck was struck with the idea that if stramonium disturbs the senses and produces mental derangement in persons who are healthy, it might very easily be administered to maniacs, for the purpose of restoring the senses, by effecting a change of ideas.

"The Danish physician Stahl has, above all other writers, expressed his conviction on this head most unequivocally: he speaks in the following terms. 'The received method in medicine, of treating diseases by opposite remedies,—that is to say, by medicines which are opposed to the effects they produce (*contraria contrariis*),—is completely false and absurd. I am persuaded, on the contrary, that diseases are subdued by agents which produce a similar affection (*similia similibus:*) burns by the heat of a fire to which the parts are exposed; the frostbite by snow or icy cold water; and inflammations and contusions by spirituous applications. It is by these means I have succeeded in curing a disposition to rigors, by using very small doses of sulphuric acid, in cases where a multitude of absorbing powders had been administered to no purpose.'

"Thus far the great truth has more than once been approached by physicians. But a transitory idea was all that presented itself to them: consequently, the indispensable reform which ought to have taken place in the old school of therapeutics, to make room for the true curative method, and a system of medicine at once simple and certain, has, till the present day, not been effected." (P. 99.)

It is curious that the two errors in the above quotation from Hippocrates, of κὰτ' for κατ', and of ἔπετος for ἔμετος, exist also in the German edition of 1824. On looking into the same book, we find that it was not rigors, but acidity of the stomach (*Magensäure*), which Stahl cured with sulphuric acid: rigors would be nothing to the purpose.

Having passed through the porch, we now enter the temple itself, and are immediately saluted with the following laconic aphorism: § 1. "The first and sole duty of the physician is to restore health to the sick: this is the true art of healing." (P. 103.) To which our author has appended a quaint note, informing us what we ought not to do.

"His mission is not (as many physicians (who wasting their time and powers in the pursuit of fame,) have imagined it to be, that of inventing systems by stringing together empty ideas and hypotheses upon the immediate essence of life, and the origin of disease, in the interior of the human economy; nor is it that of continually endeavouring to account for the morbid phenomena with their nearest cause, (which must for ever remain concealed,) and confounding the whole in unintelligible words and pompous observations, which make a deep impression on the minds of the ignorant, while the patients are left to sigh in vain for relief. We have already too many of these learned reveries which bear the name of medical theories, and for the inculcation of which even special professorships have been established. It is high time that all those who call themselves physicians should cease to deceive suffering humanity with words that have no meaning, and begin to act,—that is to say, to afford relief, and cure the sick in reality."

We are to endeavour to ascertain the cause of the disease, and the *ensemble* of the symptoms, and farther than this we cannot go; for, though every malady presupposes some change in the interior of the human frame, our understandings only permit us to form a vague and dark conception of this change from a view of the morbid symptoms, which are the sole guide we have to rely upon, except in cases that are purely surgical." He who can succeed in removing the whole group of symptoms has cured the disease; whereas, some people try to knock them on the head, one by one; a method which "has very justly excited universal contempt." Now plain experience shows that "the particular medicine whose action upon persons in health produces the greatest number of symptoms resembling those of the disease which it is intended to cure, possesses also in reality (when administered in convenient doses) the power of suppressing, in a radical, prompt, and permanent manner, the morbid symptoms; that is to say, (§ 8, 10,) the whole of the existing disease." (P. 116.) This therapeutic law of nature being an established fact, our author attaches no value to any theoretic explanation that may be given of it; yet he thinks it probable that the medicinal disease being analogous, but rather more intense, usurps the place of the original malady, and being of such a nature as to be easily subdued by the vital powers, is likewise extinguished in its turn. It is not in the power even of nature herself to cure an existing disease by one that is dissimilar; but, when she cures, she cures homœopathically. If the diseases are dissimilar, we may suppose three possible cases:

"If the two dissimilar diseases which meet together in the human body have an unequal power, or if the oldest of them is stronger than the other, the new disease will be repulsed from the body by that which existed before it, and will not be able to establish itself there. Thus a person already afflicted with a severe chronic disease, will never be subject to an attack of autumnal dysentery or any other slight epidemic. According to Larry, the plague peculiar to the Levant never breaks out in places where scurvy prevails, nor does it ever infect those who labour under dartrous diseases. According to Jenner, the rickets prevent vaccination from taking effect, and Hildebrand informs us that persons suffering under phthisis are never attacked with epidemic fevers, except when the latter are extremely violent." (P. 122.)

Instead of "unequal," it should be "equal:" this is a mistake of the translator's. Or, secondly, the new and dissimilar disease may be more powerful than the old one.

"If the new disease, which is dissimilar to the old, be more powerful than the latter, it will then cause its suspension until the new disease has either performed its own course or is cured; but then the old disease reappears. We are informed by Tulpius that two children having contracted tenia, ceased to experience any further attacks of epilepsy to which they had till then been subject; but as soon as the eruption of the head was removed they were again attacked as before. Schœpf saw the itch disappear when scurvy manifested itself, and return again after the cure of the latter disease. A violent typhus has suspended the progress of phthisis with pulmonary abscess, which resumed its march immediately after the cessation of the typhoid disease. When madness manifests itself during a pulmonary disease, it effaces the phthisis with all its symptoms; but when the mental alienation ceases, the pulmonary disease again rears its head and kills the patient. Where the measles and the small-pox exist together, and have both attacked the same infant, it is usual for the measles which have already declared themselves, to be arrested by the small-pox which bursts forth, and not to resume their course until after the cure of the latter; on the other hand, Manget has also seen the small-pox, which had fully developed itself after inoculation, suspended during four days by the measles which intervened, and after the desquamation of which, it revived again to run its course. The eruption of measles on the sixth day after inoculation has been known to arrest the inflammatory operation of the latter, and the small-pox did not break out until the other exanthema had accomplished its seven days' course. In an epidemic, the measles broke out among several patients four or five days after inoculation, and retarded, until their entire disappearance, the eruption of the small-pox which subsequently proceeded in a regular manner. The true scarlet fever of Sydenham, with angina, was arrested on the fourth day by the manifestation of cow-pock, which went through its natural course; and not before its termination did the scarlet fever manifest itself again. But as these two diseases appear to be of equal force, the cow-pock has likewise been seen to suspend itself on the eighth day by the eruption of genuine scarlatina, and the red areola was effaced until the scarlatina had terminated its career; at which moment the cow-pock resumed its course and terminated regularly. The cow-pock was on the point of attaining to its state of perfection on the eighth day when measles broke out, which immediately rendered it stationary, and not before the desquamation of which, did it resume and finish its course; so that, according to the report of Kortum, it presented on the sixteenth day the aspect which it usually wears on the tenth. The vaccine virus has been known to infect the system even where the measles had already made their appearance, but it did not pursue its course until the measles had passed away; for this we have also the authority of Kortum.

"I have myself had an opportunity of seeing a parotid angina disappear immediately after the development of the cow-pock. It was not till after the cow-pock had terminated, and the disappearance of the red areola of the vesicles, that a great swelling attended with fever, manifested itself in the parotid and sub-maxillary glands which ran its ordinary course of seven days.

"It is the same in all diseases that are dissimilar; the stronger one suspends the weaker, except in cases where they blend together, which rarely occurs in acute diseases; but they never cure each other reciprocally." (P. 123.)

Or, thirdly, the two may form a kind of alliance.

"Or it sometimes occurs that the new disease, after having acted for a considerable period upon the system, joins itself finally to the old one, presenting together a complicated form of disease, but in such a manner that each of them notwithstanding occupies a particular region of the economy, installing itself in those organs with which it sympathises, and abandoning the others to the diseases that are dissimilar. Thus a venereal affection may turn to one that is psoric, and *vice versa.* These two diseases being dissimilar, they are incapable of annihilating or curing each other. Venereal symptoms are effaced and suspended, in the first instance, as soon as a psoric eruption commences; but, in the progress of time, the venereal affection being at least quite as powerful as the psoric, the two unite together, that is to say, each seizes merely upon those parts of the organism that are appropriate to it individually, by which the patient is rendered worse, and the cure more difficult than before. In a case where two contagious acute diseases meet together, bearing no analogy to each other (such as, for example, the small-pox and the measles) one of them ordinarily suspends the other as before stated. However, there have been some extraordinary, instances in violent epidemic diseases, where two dissimilar acute maladies have simultaneously attacked the body of the same individual, and became, so to express it, complicated for a short time. In an epidemic where the small-pox and the measles reigned together, there were about three hundred cases in which one of these maladies suspended the other and in which the measles did not break forth until twenty days after the eruption of the small-pox, and the latter till from seventeen to eighteen days after that of the measles, that is to say, until after the first disease had ran its entire course; but there was a single instance in which P. Russell met with these two dissimilar maladies simultaneously in the same patient. Rainey saw the small-pox and the measles together in two little girls; and J. Maurice remarks that he never met with more than two instances of this kind in the whole course of his practice. Similar examples may be found in Ettmüller and a few other writers. Zencker saw the cow-pock pursue its course in a regular manner conjointly with measles and purpura, and Jenner likewise observed it pursue its course tranquilly in the midst of a mercurial treatment directed against the venereal disease." (P. 129.)

This result, however, more frequently occurs from a prolonged use of violent non-homœopathic remedies.

Nature has a few, but only a few, diseases, which she uses as homœopathic remedies: thus the small-pox, so famous for the violence and number of its symptoms, has cured a multitude of diseases that were characterized by symptoms like its own; and even vaccination has done the same.

" Violent ophthalmia, extending even to the loss of sight, is one of the most ordinary occurrences in the small-pox; whereas Dezoteux and Leroy have each reported cases of chronic ophthalmia which were cured in a perfect and permanent manner by inoculation.

" A case of blindness of two years standing brought on by the metastasis of tenia, was, according to Klein, perfectly cured by the small-pox. How often has the small-pox cured deafness and oppressed respiration? J. F. Closs has seen it cure both these affections when it had reached its highest state of intensity.

" Considerable enlargement of the testicle is a frequent symptom in small-pox, and according to Klein it has been known to cure homœopathically a large hard swelling of the left testicle, the consequence of a contusion. Another observer has seen it cure a similar swelling of the testicle.

" Dysentery is one of the bad symptoms which occur in smallpox, for this reason it cures the former disease homœopathically as in a case reported by F. Wendt.

"The small-pox which comes on after vaccination destroys the latter immediately, and does not permit it to arrive at perfection, both because it is more powerful than the cow-pock and bears a close resemblance to it. By the same reason, when the cow-pock approaches to its term of maturity, it diminishes and softens in a very great degree the small-pox which has just broken out, and causes it to assume a milder form, as witnessed by Mühry and many others.

"The cow-pock, in addition to the vesicles which protect from small-pox, excites also a general cutaneous eruption of another kind. This exanthema consists of sharp-pointed pimples, usually small, seldom large and suppurating, dry, resting upon a small red areola, frequently interspersed with small round spots of a red colour, and sometimes attended with severe itching. In many children it precedes by several days the appearance of the red areola of the cow-pock. But most often it manifests itself afterwards, and disappears in a few days, leaving small hard red spots on the skin. It is by reason of this other exanthema, and the analogy which it bears to the same, that the cow-pock, the moment it takes, removes in a permanent manner those cutaneous eruptions which exist in some children, and which are often troublesome and of long standing. This has been attested by numerous observers.

"Vaccination, whose special symptom is a swelling of the arm, cured, after its eruption, the tumefaction of an arm that was half paralysed.

"The vaccine fever which takes place at the period of the formation of the red areola has, according to the information of Hardège, cured two cases of intermittent fever homœopathically, which confirms the remark formerly made by J. Hunter, that two fevers (or diseases that are similar) can never exist together in the body." (P. 135.)

By tenia, our translator means tinea capitis.

But, while nature is very limited in these homœopathic resources, the physician possesses them in the greatest abundance, and, after such evidence and examples, says Hahnemann, it is impossible that he can use any others; for, to use allopathic remedies is quite absurd, and the common antipathic or enantiopathic medicines palliate the symptoms for a moment, and then they return with increased intensity. But here we come to the weakest point in the homœopathic theory: our author is obliged to allow that there are cases where palliatives are admissible.

"It is merely in urgent and dangerous cases, or in diseases that have just broken out in persons who were previously in health, such, for example, as in asphyxia by lightning, suffocation, freezing, drowning, &c. that it is either admissible or proper in the first instance at least to re-animate the feeling and irritability by the aid of palliatives, such as slight electric shocks, injections of strong coffee, stimulating odours, warmth, &c. As soon as physical life is re-animated, the action of the organs that support it resumes its regular course, as is to be expected from a body that was in the full enjoyment of health previous to the accident. Under this head are also included the antidotes to several poisons, such as alkalis against mineral acids, liver of sulphur against metallic poisons, coffee, camphor (and ipecacuanha) against poison by opium, &c." (P. 157.)

But, if allowable in these very urgent cases, where life is immediately saved by them, why not in cases where the danger, though great, is not quite so urgent?

After some less important matter, we come to the startling assertion that

" Psora is the sole true and fundamental cause that produces all the other countless forms of disease which, under the names of nervous debility, hysteria, hypochondriasis, insanity, melancholy, idiocy, madness, epilepsy, and spasms of all kinds, softening of the bones, or rickets, scoliasis and cyphosis, caries, cancer, fungus hematodes, gout, hemorrhoids, yellow jaundice and cyanosis, dropsy, amenorrhea, gastrorrhagia, epistaxis, hemoptysis, hematuria, metrorrhagia, asthma and suppuration of the lungs, impotency and barrenness, megrim, deafness, cataract and amaurosis, gravel, paralysis, loss of sense, pains of every kind, &c. appear in our pathology as so many peculiar, distinct, and independent diseases." (P. 168.)

On first perusing this sentence, it would appear to many persons that our learned author had adopted a nosology even more loose and comprehensive than that of the very vulgar, who are apt, under the denominations of scurvy, inward affection, &c., to comprehend a whole host of diseases, yet not quite so many as psora seems to cover. But a little farther on we find that "the homœopathic physician, at every chronic disease (psoric) that he is called upon to treat, ought not to be less careful than before in seizing upon the perceptible symptoms, and everything that is connected with them; for it is no more possible in these diseases than in others to obtain a real cure without particularizing each individual case in a rigorous and absolute manner." (P. 173.) So that psora is merely an awkward synonyme for the cachexiæ, or rather for chronic disease in general, and not a malady to be treated blindly with some one specific: on the contrary, our author is for investigating all the symptoms with indefatigable minuteness, and gives long directions as to the manner of examining patients. The following remark appears to us very acute:

"On the other hand, the patients are so accustomed to their long sufferings that they pay little or no attention to the lesser symptoms, which are often very characteristic of the disease and decisive in regard to the choice of the remedy; they look upon them as though they were in a manner belonging to their physical state, and constituted a part of that health the real sentiment of which they had forgotten during the fifteen or twenty years their sufferings have endured, and never entertain a suspicion that there can be any connexion between these symptoms and the principal disease." (P. 183.)

Passing over much curious matter, which our limits compel us to leave untouched, we come to the other essential point of Dr. Hahnemann's theory—the minuteness of the doses. No reasoning, however ingenious, says our author, can show how small they ought to be.

"It would be absurd to bring forward as an objection the large doses used in ordinary medicine, which are not applied to the suffering parts themselves but merely to those not attacked by the disease. This would be no argument against the weakness of the doses which pure experiments have proved to be necessary in homœopathic treatment." (P. 292.)

The next section informs us of the principle by which we know whether a homœopathic remedy has reached the highest possible degree of attenuation, which is at the same time the acme of perfection.

"It has been fully proved by pure experiments that when a disease does not evidently depend upon the impaired state of an important organ, even though it were of a chronic nature and complicated, and due care has been taken to remove from the patient all foreign medicinal influence, the dose of the homœopathic remedy can never be sufficiently small so as to be inferior to the power of the natural disease, which it can extinguish and cure, provided it retains the degree of energy necessary to excite symptoms rather more intense than its own immediately after it is administered." (P. 293.)

Mr. Devrient and Dr. Stratten ought to have found out between them, that "more intense than its own" makes nonsense of the sentence: it should have been "more intense than those of the disease;" and so it stands in our Hahnemann, "So lange sie noch einige, obschon geringe Erhöhung ihrer Symptome über die ihr ähnliche Krankheit gleich nach ihrer Einnahme zu verursachen im Stande ist." [p. 268, Ed. 1824.]

The smaller the dose the better, provided it fulfils this indication, even though the diminution should be so great as to appear incredible to the coarse material comprehension of every-day physicians: their babble (says Hahnemann) must be struck dumb when infallible experience pronounces its sentence. They may learn, too, from mathematicians, that the smallest fraction of matter is still something, and can never be reduced by subdivision to nothing; and, if they are capable of instruction, they may learn from those skilled in physics that enormous powers exist which have no weight at all, such as heat and light, which are therefore lighter than the smallest homœopathic dose.*

The scale by which the doses are to be regulated is explained in the following section:

"The effects of a dose are by no means diminished in the same proportion as the quantity of the medicinal substance is attenuated in the homœopathic practice. Eight drops of a tincture taken at once do not produce upon the human body four times the effect of a dose of two drops; they merely produce one that is nearly double. In the same manner the single drop of a mixture, composed of one drop of a tincture and ten of a liquid, void of all medicinal properties, does not produce ten times the effect that a drop ten times more attenuated would produce, but merely an effect that is scarcely double. The progression continues according to this law, so that a single drop of a dilution, attenuated in the highest degree, ought, and does in fact, produce a very considerable effect." (P. 297.)

To this is appended a note, giving a more detailed explanation; a note which Mr. Devrient has misunderstood and mangled as never note was misunderstood and mangled before. Hahnemann says, that if a drop of a mixture containing $\frac{1}{10}$ of a grain of a medicine produces an effect $= a$, then a drop of a weaker one, containing $\frac{1}{100}$ of a grain, will produce an effect $= \frac{a}{2}$; if it contains $\frac{1}{10000}$ of a grain, the effect will be $= \frac{a}{4}$, and so on; which may be put into a tabular form thus, to make it clearer:

| Quantity of medicine contained in the mixtures. | Effect produced by a drop. |
|---|---|
| $\frac{1}{10}$ of a grain | $a$ |
| $\frac{1}{100}$ | $\frac{a}{2}$ |
| $\frac{1}{10000}$ | $\frac{a}{4}$ |
| $\frac{1}{100,000,000}$ | $\frac{a}{8}$ |

So that each dose of the medicine is the square root† of the preceding one, while each effect is one half of the preceding

* The translator, at p. 294, *note*, has mistaken Physikern for physicians: it means those skilled in physics, (*physici.*)

† Or the square, if the dose be considered merely as a fraction.

one, the quantity of mixture taken always being the same. But our translator and commentator, not understanding these matters, have put Hahnemann's light under a bushel, in the following manner:

"Suppose, for example, that one drop of a mixture containing the tenth of a grain of any medicinal substance produces an effect $=a$; a drop of another mixture containing merely an hundredth part of a grain of this same substance will only produce an effect $=\frac{a}{2}$; if it contains a ten-thousandth part of a grain of medicine, the effect will be $=\frac{a}{4}$; if a millionth, it will be $=\frac{a}{8}$; and so on progressively, to an equal volume of the doses, the effects of the remedy on the body will merely be diminished about one half each time that the quantity is reduced nine tenths of what it was before. I have often seen a drop of the tincture of nux vomica at the decillionth degree of dilution produce exactly half the effect of another at the quintillionth degree, when I administered both one and the other to the same individual, and under the same circumstances." (P. 297.)

The latter part of the note has been translated correctly, but the reader must not imagine, as Dr. Stratten does, (note xv.) that quintillionth means the five millionth part: it means the $\frac{1}{\overline{1{,}000{,}000}|^5}$th part; for quintillion does not signify five millions, but a million raised to the fifth power; and decillionth, which Dr. S. (Note vi.) explains to mean "a degree of dilution common in homœopathic practice," signifies the $\frac{1}{\overline{1{,}000{,}000}|^{10}}$th part: so that, in the above note of Hahnemann, the decillionth and quintillionth of a grain mixtures stand to one another in the same relation as those respectively containing $\frac{1}{10}$ and $\frac{1}{100}$ of a grain.

In another place, our translator (or his commentator,) has endeavoured "to explain the degree of dilution substances are capable of." But they have made sad work of it! Having no original in this case to guide us, we are compelled to leave the passage to our readers to clear up and understand as well as they can.

"One grain of nitrate of silver dissolved in 1560 grains of distilled water, to which were added two grains of muriatic acid, a grey precipitate of chloride of silver was evident in every part of the liquor. One grain of iodine dissolved in a drachm of alcohol and mixed with the same quantity of water as in the preceding experiment, to which were added two grains of starch dissolved in an ounce of water, caused an evident blue tint in the solution: in these experiments the grain of the nitrate of silver and iodine must have been divided into $\frac{1}{15360}$ of a grain." (P. vii.)

We have now given a sketch, though we fear an imperfect one, of the theory of that great and ingenious physician, Dr. Hahnemann; and it will naturally be expected that we should offer our opinion as to its truth. If the practicability of his method of healing rested on his own authority alone, we should of course rank it with those notable discoveries of which our medical books are full; as, when we are informed, for example, that, in the hands of Dr. A., of B., the flowers of C. would infallibly cure the disease D., but always failed to do so in the hands of every one else, we reasonably conjecture that Dr. A. picked out the slightest cases for the exhibition of the powers of his new remedy. But Dr. Hahnemann's system is not open to this objection at least, as it has been tried with success, on the largest scale, by other physicians, in other towns. We are compelled to allow, therefore, either that a drop of mixture containing $\frac{1}{1,000,000^{10}}$ of a grain of aconite may have very decided effects on the morbid animal economy, or that cases will get well under the influence of well-regulated diet and regimen, (for on these Dr. H. lays the greatest possible stress,) which have hitherto been considered incurable by these means alone. The latter supposition appears by far the most probable.

Dr. Stratten has tacked to this translation some of the poorest notes in existence. It is impossible to say for whose use they can be designed: certainly not for persons qualified to read or appreciate Hahnemann. The first four may show how flat and trite these annotations are.

"NOTE I, page 2.—DYNAMIC, from δυναμις, *force*, used to denote vital force.

"Hahnemann presumes that the origin of disease acting on the animal economy, and effecting general changes therein, is to be removed by medicinal agents which operate upon the morbid essence, and consequently upon the entire system.

"NOTE II. p. 2.—NORMAL, from *norma*, form or pattern; the state of the body in those who died a natural death, or according to the law of nature, the parts having undergone no change from the healthy condition.

"NOTE III. p. 3.—FACTORS, from *facio*, to make. This term applies to the individual parts of the nervous system, which, as a whole, is the source of sensibility, irritability, and nutrition.

"NOTE IV. p. 4.—CAUSAL INDICATION. Causal, from *causa*, relating to causes; indication, from *indico*, to point out. Indication is of four kinds, vital, preservative, curative, and palliative; 1st, as it directs what is to be done to continue life; 2d, cutting off the cause of an approaching disease; 3d, curing it whilst it is actually present; 4th, or lessening its effects.

"Experience corresponds with the doctrine of the author, and proves that a practice founded on causal indication frequently fails in curing disease." (P. 309.)

Dr. Stratten, indeed, is rather fond of teaching the etymology of the technical terms in physic; an office for which he is most eminently disqualified; *e. g.*

"Hæmaturia, from αἰμα, blood, and τυρευω, to mix together; the medicinal term for discharge of blood from the urethra mixed with the urine." (P. 324.)

Is there any one but the commentator who does not know that Hæmaturia is derived from αἷμα, and ὀυρεῖν, to make water? Τυρευω, by the way, is an imaginary word.

The names of plants, like the plants themselves, have so often been brought from the East, that, if we wish to trace their etymologies to the fountain-head, we must look for them in the languages of Arabia, Persia, or Hindoostan. Dr. S., on the contrary, has indulged in such plays on words as the following, which have very much the air of being imitations of Swift's jocose derivations of Ajax from *a jakes*, and so on:

"Daphne, from Δα, to burn, and φωνω, to make a sound; because the leaves crackle when burning." (P. 319.)—Where was Δα picked up?

"Senna. *Name*, from *sanare*, to cure." (P. 325.)

We are sorry to disturb Dr. S. in his jest; but senna is an Oriental word: in Arabic, for instance, it is Sunā, as we learn from Ainslie.

"Datura Stramonium, name from *daturus*, because it is given as a narcotic, and στρυχνομανικον, from its causing madness." We assure our readers that this inimitable bit of drollery is copied *verbatim et literatim* from Dr. Stratten's book, p. 328 *et seq.*

We will not mention the true etymon of Datura, (from Dhétoora, its Hindoostanee name,) lest we should be accused of throwing cold water on the best attempt of the kind that has been made since the days of the dean of St. Patrick's; but we will recommend tyros, should they be asked, in their examinations, for the derivations of senna, datura, &c., not to copy our commentator's waggery; for what might pass for a jest in a doctor of physic, would in them be accounted ignorance.

